# Interleukin(IL)-36α and IL-36γ Induce Proinflammatory Mediators from Human Colonic Subepithelial Myofibroblasts

**DOI:** 10.3389/fmed.2015.00069

**Published:** 2015-09-22

**Authors:** Toshihiro Kanda, Atsushi Nishida, Kenichiro Takahashi, Kentaro Hidaka, Hirotsugu Imaeda, Osamu Inatomi, Shigeki Bamba, Mitsushige Sugimoto, Akira Andoh

**Affiliations:** ^1^Department of Medicine, Shiga University of Medical Science, Otsu, Japan

**Keywords:** interleukin-1, interleukin-36, colon, myofibroblasts, inflammatory bowel disease

## Abstract

**Background:**

Interleukin (IL)-36 cytokines are recently reported member of the IL-1 cytokine family. However, there is little information regarding the association between IL-36 cytokines and gut inflammation. In the present study, we investigated the biological activity of IL-36α and IL-36γ using human colonic subepithelial myofibroblasts (SEMFs).

**Methods:**

The mRNA expression and the protein expression of target molecules in SEMFs were evaluated using real-time polymerase chain reaction and enzyme-linked immunosorbent assay, respectively. The intracellular signaling of IL-36 cytokines was analyzed using Western blot analysis and small interfering RNAs (siRNAs) specific for MyD88 adaptor proteins (MyD88 and IRAK1) and NF-κB p65.

**Results:**

IL-36α and IL-36γ significantly enhanced the secretion of IL-6 and CXC chemokines (CXCL1, CXCL2, and CXCL8) by SEMFs. The combination of IL-36α/γ and IL-17A or of IL-36α/γ and tumor necrosis factor-α showed a synergistic effect on the induction of IL-6 and CXC chemokines. The mRNA expression of proinflammatory mediators induced by IL-36α and/or IL-36γ was significantly suppressed by transfection of siRNA for MyD88 or IRAK1. Both inhibitors of mitogen activated protein kinases and siRNAs specific for NF-κBp65 significantly reduced the expression of IL-6 and CXC chemokines induced by IL-36α and/or IL-36γ.

**Conclusion:**

These results suggest that IL-36α and IL-36γ contribute to gut inflammation through the induction of proinflammatory mediators.

## Introduction

Inflammatory bowel disease (IBD), which includes ulcerative colitis (UC) and Crohn’s disease (CD), is characterized by chronic inflammation in the gastrointestinal tract (1–3). A dysfunction of the host immune response to dietary factors and/or the gut microbiota has been reported to play an important role in the pathogenesis (4–9).

IL-36 is a novel IL-1 cytokine family member and consists of IL-36α, IL-36β, IL-36γ, IL-38, and IL-36 receptor antagonist (IL-36Ra) (10–13). IL-36 cytokines were initially identified through the use of DNA database searches for homologs to IL-1 (14). IL-36 cytokines bind to the IL-36 receptor (IL-36R) and use the IL-1 receptor accessory protein (IL-1RAcp) as a co-receptor (14, 15), leading to the activation of NF-κB and MAPK pathways (10, 14). Recent studies showed that IL-36 cytokines play an important role in the pathogenesis of psoriasis (16–20), rheumatoid arthritis (21–23) or pulmonary disease (24, 25). However, there are few reports regarding the function of IL-36 in gut inflammation.

Human colonic subepithelial myofibroblasts (SEMFs) are located immediately subjacent to the basement membrane in normal intestinal mucosa, juxtaposed against the bottom of epithelial cells (26, 27). We have previously reported that human colonic SEMFs play an important role in chronic inflammation and wound healing in the intestine and contribute to the pathogenesis of IBD (26, 27).

In the present study, to explore the potential role of IL-36 cytokines in gut inflammation, we investigated the biological functions and the signal transduction of IL-36α and IL-36γ in human colonic SEMFs.

## Materials and Methods

### Reagents

Recombinant human IL-36α and TNF-α were purchased from R&D Systems Inc. (Minneapolis, MN, USA). Human IL-36γ and IL-17A were purchased from Pepro Tech Inc. (Rocky Hill, NJ, USA). Inhibitors of MEK1 that is upstream of p42/44 ERK1/2 (PD98059 and U0126) and an inhibitor of the p38 MAPK (SB203580) were purchased from Cell Signaling Technology (Tokyo, Japan). The siRNAs specific for MyD88, IRAK1, and NF-κBp65 and a control siRNA were purchased from Santa Cruz Biotechnology (Santa Cruz, CA, USA).

### Human colonic subepithelial myofibroblasts

Primary colonic SEMFs cultures were prepared from normal colonic tissues according to the method reported by Mahida et al (28). Normal colonic tissues were obtained from surgical specimens (>5 cm from the tumor margin) of patients undergoing a partial colectomy for carcinoma. The cells were cultured in DMEM (Nacalai Tesque Inc., Kyoto, Japan) containing 10% fetal bovine serum. All culture media were supplemented with 50 U/ml penicillin and 50 μg/ml streptomycin. Over 98% of the cells were immune-positive for α-SMA, a marker of myofibroblasts. The studies were performed on passages two to six of myofibroblasts isolated from four different resection specimens. All experiments and all protocols were approved by the ethical committee of Shiga University of Medical Science. Written informed consent was obtained from all subjects in compliance with the Declaration of Helsinki.

### Real-time PCR analysis

The mRNA expression in the samples was assessed using real-time polymerase chain reaction (PCR) analyses. The real-time PCR was performed using the Light Cycler 480 system (Roche Applied Science, Tokyo, Japan) and the reagent SYBR Premix Ex Taq II (TAKARA, Otsu, Japan). The oligonucleotide primers used in this study were as follows: IL-6 sense primer, GGTACATCCTCGACGGCATCT; anti-sense primer, GTGCCTCTTTGCTGCTTTCAC (NCBI Gene ID: 000600); CXCL8 sense primer, AGGGTTGCCAGATGCAA TAC; anti-sense primer, GCAAACCCATTCAATTCCTG (NCBI Gene ID: 000584); CXCL1 sense primer, TGTTTGAGCAT CGCTTAGGA; anti-sense primer, GATCTCATTGGCCATTT GCT (NCBI Gene ID: 001511); CXCL2 sense primer, GGATT GCGCCTAATGTGTTT; anti-sense primer, CACTGGCCATT TTCTTGGAT (NCBI Gene ID: 002089); β-actin sense primer, TGACCCAGATCATGTTTGAGACCT; and β-actin anti-sense primer, CCACGTCACACTTCATGATGATGGAG. The data were normalized versus β-actin mRNA.

### Enzyme-linked immunosorbent assay

The concentrations of CXCL1, CXCL8, and IL-6 in the culture medium were quantified using the human Quantikine ELISA Kit (R&D Systems). CXCL2 was quantified using the Human Omnikine ELISA Kit (Assay Biotech). All procedures were performed according to the manufacturer’s protocol.

### Western blot analysis

Human colonic SEMFs were exposed to IL-36α (100 ng/ml) or IL-36γ (100 ng/ml) for pre-determined periods. The stimulated cells were lysed in a protein extraction buffer (20 mM Tris-HCl, 2 mM EDTA, 2 mM EGTA, 150 mM NaCl, 400 mM sodium fluoride, 4 mM sodium orthovanadate, 1% NP-40 Non-idet, containing complete mini). Western blots were then performed according to a method previously described (29). Antibodies against phosphorylated and total MAPKs, IκBα and glyceraldehyde-3-phosphate dehydrogenase (GAPDH), were purchased from Cell Signaling Technology (Tokyo, Japan). The signal was detected using the enhanced chemiluminescence western blotting system (GE Healthcare, Little Chalfont, UK).

### Small interference RNA experiments

Human colonic SEMFs were transfected with siRNA using Lipofectamine RNAiMAX (Invitrogen) according to the manufacturer’s instructions. Briefly, cells were cultured in complete medium in the presence of a mixture of an RNAi duplex and Lipofectamine RNAiMAX for 24 h, and were then stimulated with or without IL-36α or IL-36γ for 12 h.

### Statistical analysis

The statistical significance of the differences between groups was determined using Student’s *t*-test. Data are expressed as means ± SD (*n* = 4). Differences resulting in *P*-values <0.05 were considered to be statistically significant.

## Results

**Figure 1:**
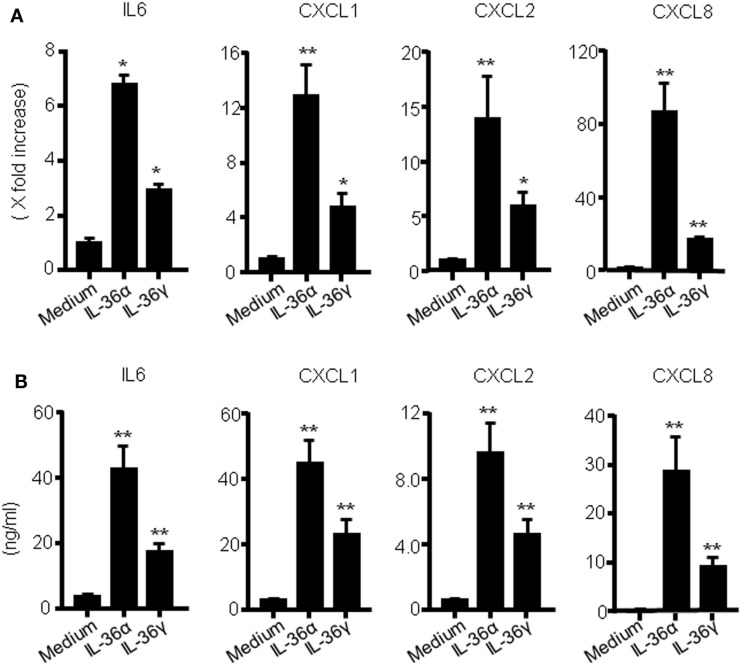
**IL-36α and IL-36γ induce IL-6 and CXC chemokine mRNA expression in human colonic SEMFs**. **(A)** SEMFs were stimulated with IL-36α (100 ng/ml) or IL-36γ (100 ng/ml) for 24 h. The mRNA expression for IL-6 and CXC chemokines (CXCL1, CXCL2, and CXCL8) was evaluated using real-time PCR. The mRNA expression for IL-6 and chemokine was converted to a value relative to β-actin mRNA expression and presented as fold-increase relative to the results for medium alone (no stimulation). Data are expressed as means ± SD of four independent experiments. *P < 0.05, **P < 0.01; significant differences from the values for medium alone. **(B)** SEMFs were incubated with IL-36α (100 ng/ml) or IL-36γ (100 ng/ml) for 24 h. The protein levels of IL-6 and CXC chemokines (CXCL1, CXCL2, and CXCL8) in the culture medium were determined using ELISA. Data are expressed as means ± SD of four independent experiments. *P < 0.05, **P < 0.01; significant differences from the values for medium alone.

To investigate the function of IL-36 cytokines in human colonic SEMFs, the cells were stimulated with IL-36α (100 ng/ml) or IL-36γ (100 ng/ml) for 24 h, and the mRNA expression of IL-6 and CXC chemokines (CXCL1, CXCL2, and CXCL8) was evaluated using real-time PCR. The secretion of IL-6 and CXC chemokine proteins was also determined using ELISA. As it has been reported that IL-36α, IL-36β, and IL-36γ can be activated by the removal of N-terminal residues, we used spliced recombinant IL-36 cytokines (30). As shown in Figures 1A,B, both IL-36α and IL-36γ induced a significant increase in the mRNA and protein expression of these proinflammatory mediators compared to the medium control. These results indicate that IL-36α and IL-36γ are strong inducers of proinflammatory mediators in human colonic SEMFs.

**Figure 2:**
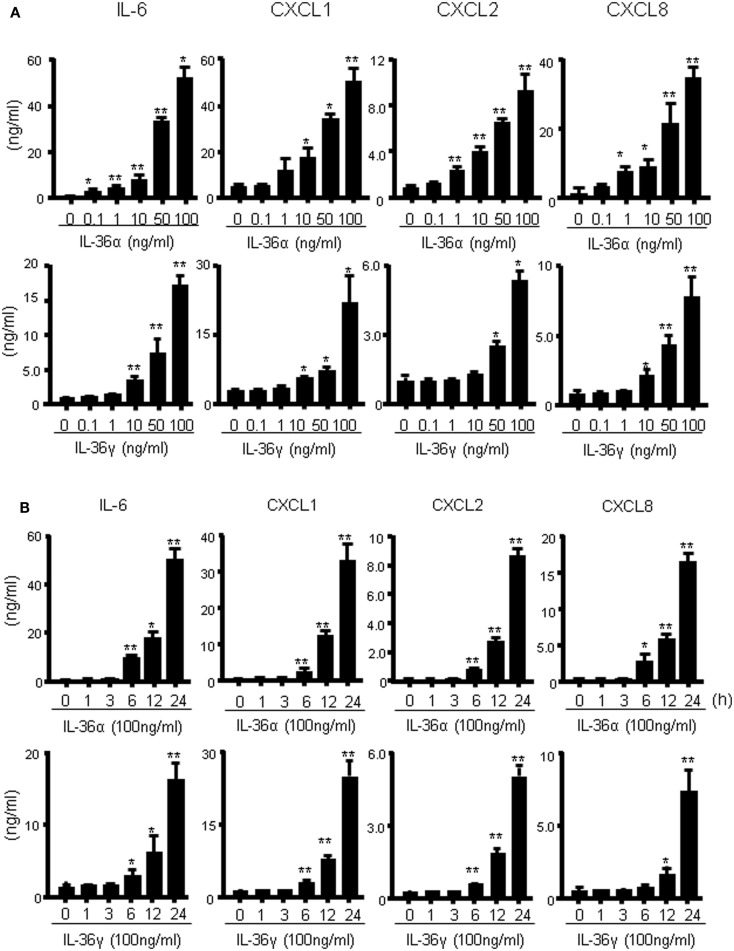
**IL-36α and IL-36γ dose- and time-dependently induce IL-6 and CXC chemokines in human colonic SEMFs**. **(A)** Dose-dependent effects of IL-36α or IL-36γ on IL-6 and CXC chemokines. SEMFs were stimulated for 24 h with increasing concentrations of IL-36α or IL-36γ, and the concentration of IL-6 and CXC chemokines (CXCL1, CXCL2, and CXCL8) secreted into the culture medium was determined using ELISA. Data are expressed as means ± SD of four independent experiments. *P < 0.05, **P < 0.01; significant differences from the values for medium (0 ng/ml) alone. **(B)** The kinetics of IL-6 and CXC chemokine induction by IL-36α and IL-36γ. SEMFs were stimulated with 100 ng/ml of IL-36α or 100 ng/ml of IL-36γ for pre-determined times, and the protein levels of IL-6 and CXC chemokines in the culture medium were measured using ELISA. Data are expressed as means ± SD of four independent experiments. *P < 0.05, **P < 0.01; significant differences from the values at 0 h.

We investigated the induction of proinflammatory mediators by IL-36α and IL-36γ in further detail. Human colonic SEMFs were incubated for 24 h with increasing concentrations of IL-36α or IL-36γ, and the secretion of CXC chemokines was measured using ELISA. IL-36α or IL-36γ dose-dependently induced the secretion of CXC chemokines (Figure 2A). We next evaluated the protein expression of IL-6 and CXC chemokines. Human colonic SEMFs were incubated with IL-36α (100 ng/ml) or IL-36γ (100 ng/ml) for pre-determined times and the secretion of IL-6 and CXC chemokines was measured using ELISA. As shown in Figure 2B, IL-36α and IL-36γ induced the secretion of proinflammatory mediators in a time-dependent manner (effects of IL-36α and IL-36γ at the mRNA levels were shown in Figure S1 in Supplementary Material).

**Figure 3:**
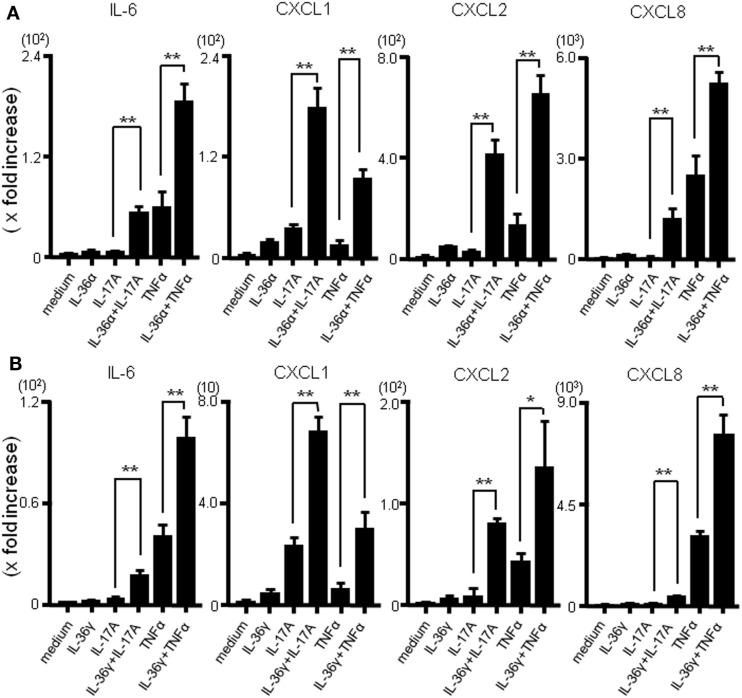
**Effects of combination of IL-36α/γ plus IL-17A or that of IL-36α/γ plus TNF-α on the induction of IL-6 and CXC chemokines**. SEMFs were stimulated with combination of IL-36α/γ plus IL-17A or that of IL-36α/γ plus TNF-α for 24 h [**(A)** IL-36α plus IL-17A or IL-36α plus TNF-α, **(B)** IL-36γ plus IL-17A or IL-36γ plus TNF-α]. The mRNA expression of IL-6 and CXC chemokines (CXCL1, CXCL2, and CXCL8) was then determined using real-time PCR. The mRNA expression for IL-6 and chemokine was converted to a value relative to β-actin mRNA expression and presented as fold-increase relative to the results for medium alone (no stimulation). Data are expressed as means ± SD of four independent experiments. *P < 0.05, **P < 0.01; significant differences from the values for IL-17A or TNF-α stimulation.

The combined effect of IL-36 and IL-17A or IL-36 and TNFα on the mRNA production of inflammatory mediators was evaluated using real-time PCR. As shown in Figure 3A, the combination of IL-36α and IL-17A or IL-36α and TNFα synergistically enhanced the mRNA expression of IL-6 and CXC chemokines. Similarly, simultaneous stimulation with IL-36γ and IL-17A or IL-36γ and TNFα synergistically upregulated the mRNA expression of IL-6 and CXC chemokines (Figure 3B).

**Figure 4:**
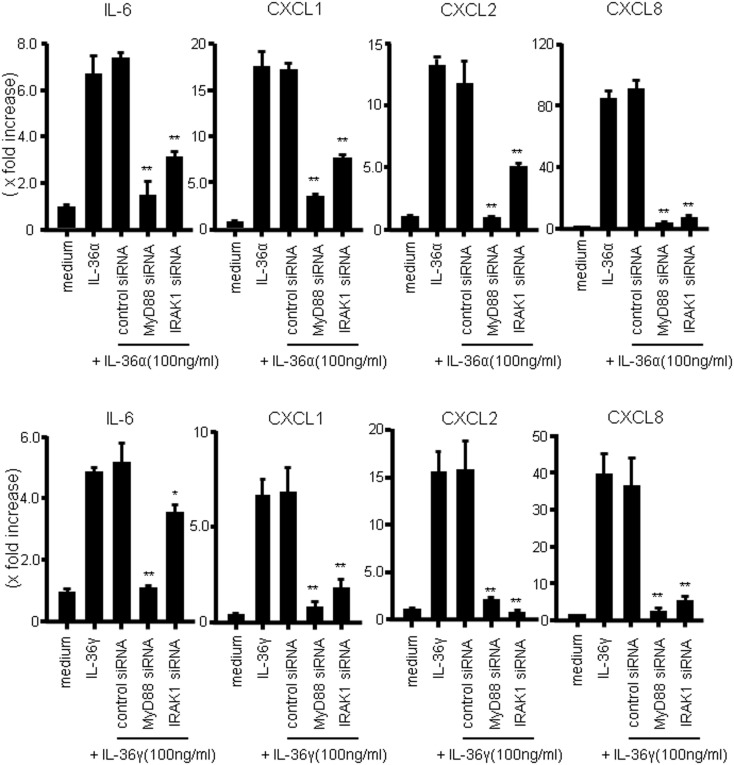
**Role of MyD88 adaptor proteins (MyD88 and IRAK1) in IL-36 signaling in human colonic SEMFs**. SEMFs were transfected with siRNA specific for the MyD88 adaptor proteins (MyD88 and IRAK1) or with control siRNA. The cells were incubated for 24 h with or without 100 ng/ml of IL-36α (upper panels) or IL-36γ (lower panels). The mRNA expression of IL-6 and CXC chemokines (CXCL1, CXCL2, and CXCL8) was then evaluated using real-time PCR. The mRNA expression for IL-6 and chemokine was converted to a value relative to β-actin mRNA expression and presented as fold-increase relative to the results for medium alone (no stimulation). Data are expressed as means ± SD of four independent experiments. *P < 0.05, **P < 0.01; significant differences from the values for IL-36α or IL-36γ stimulation.

We next investigated whether MyD88 adaptor proteins were involved in the signal transduction of IL-36α or IL-36γ. For this purpose, we employed siRNA transfection system to silence the gene expression of MyD88 adaptor proteins. As shown in Figure 4, siRNA specific for MyD88 or IRAK1, but not control siRNA, significantly suppressed the mRNA expression of IL-6 and CXC chemokines (CXCL1, CXCL2, and CXCL8) in response to IL-36α or IL-36γ [efficacy of siRNAs specific for MyD88, IRAK1, and NF-κB65 was confirmed by real-time PCR (Figure S2 in Supplementary Material)]. These findings indicated that MyD88 adaptor proteins are essential for the intracellular signaling of both IL-36α and IL-36γ for the induction of proinflammatory mediators.

**Figure 5:**
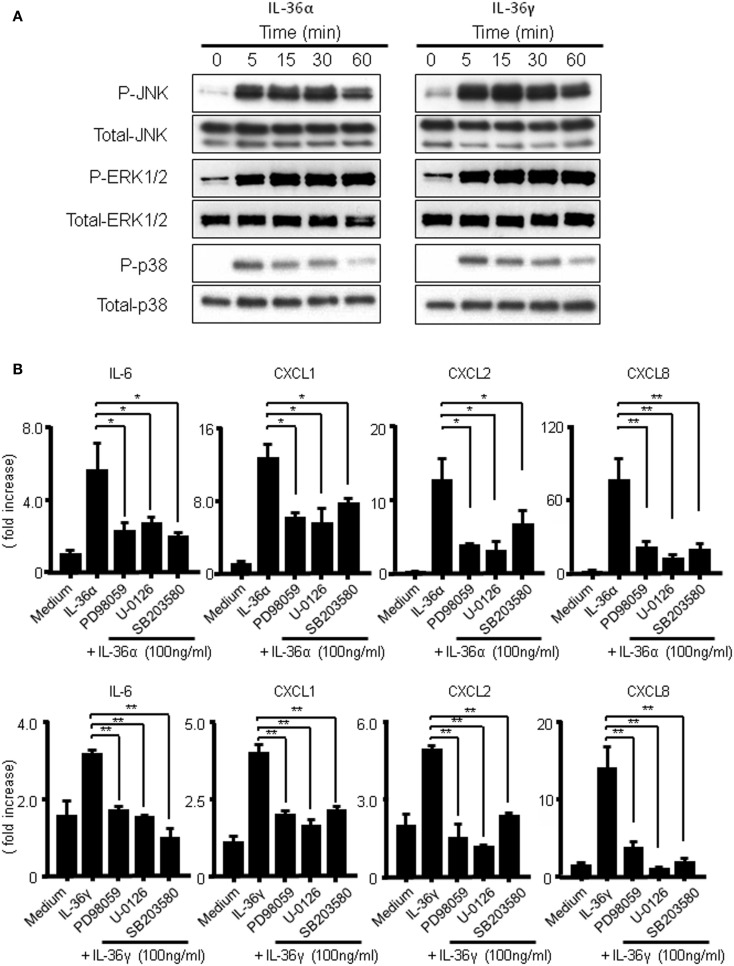
**Activation of MAPKs by IL-36α or IL-36γ in human colonic SEMFs**. **(A)** SEMFs were stimulated with or without IL-36α (100 ng/ml) or IL-36γ (100 ng/ml) for the indicated pre-determined times. Expression of phosphorylated (p-) and total MAPKs were sequentially evaluated by Western blotting. The data are representative of two independent experiments. **(B)** SEMFs were pretreated with 20 μM of a p38 MAPK inhibitor (SB203580) or an MEK1/2 inhibitor (U0126 or PD98059) for 1 h, and were then incubated with or without 100 ng/ml of IL-36α (top panels) or IL-36γ (bottom panels) for 24 h. The mRNA expression of IL-6 and chemokines was then analyzed. The mRNA expression for IL-6 and chemokines was converted to a value relative to β-actin mRNA expression and presented as fold-increase relative to the results for medium alone (no stimulation). Data are expressed as means ± SD of four independent experiments. *P < 0.05, **P < 0.01; significant differences from the values for IL-36α or IL-36γ stimulation.

It has previously been reported that IL-36 cytokines activate MAPKs and NF-κB in Jurkat cells (14). To assess whether the activations of MAPKs are involved in the induction of IL-6 and CXC chemokines (CXCL1, CXCL2, and CXCL8) by IL-36α or IL-36γ, we evaluated the phosphorylation of MAPKs in IL-36-stimulated human colonic SEMFs by Western blot analysis. As shown in Figure 5A, both IL-36α and IL-36γ rapidly induced the phosphorylation of p42/44 MAPK, p38 MAPK, and JNK within 5 min after stimulation. Next, we examined the involvement of MAPKs in the induction of IL-6 and CXC chemokines (CXCL1, CXCL2, and CXCL8) by IL-36α or IL-36γ using specific inhibitors of p38 MAPK (SB203580) and of MEK1 (PD98059 and U0126) that is directly upstream of p42/44MAPK. As shown in Figure 5B, inhibition of p38 MAPK or of p42/44MAPK significantly reduced the mRNA expression of IL-6 or CXC chemokines induced by IL-36α or IL-36γ. These results indicated that the activation of MAPKs is involved in the induction of proinflammatory mediators by both IL-36α and IL-36γ in human colonic SEMFs.

**Figure 6:**
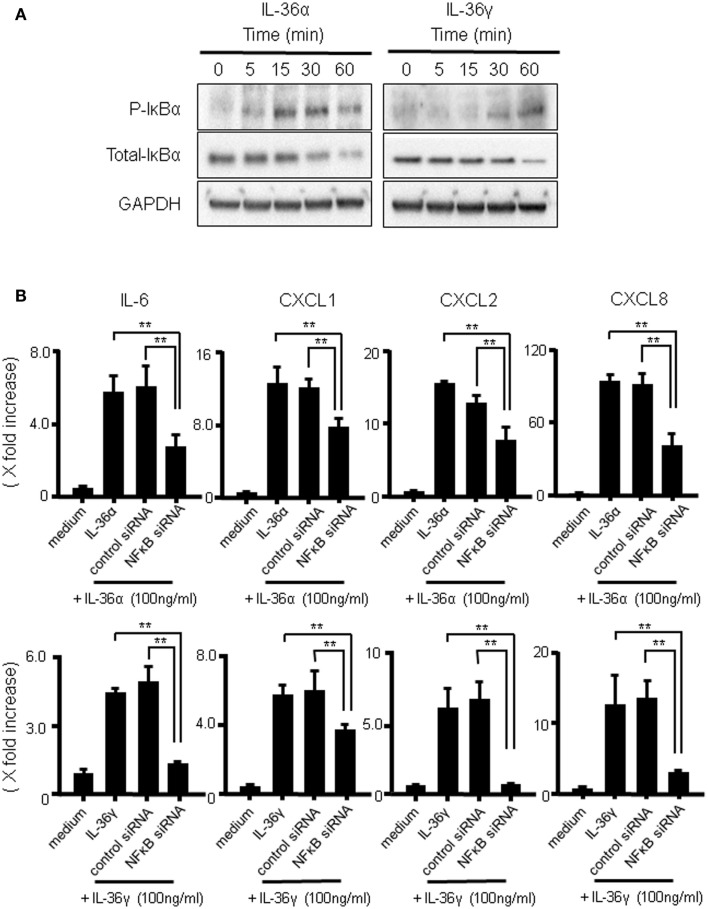
**The activation of IκBα by IL-36α or IL-36γ in human colonic SEMFs**. **(A)** SEMFs were stimulated with 100 ng/ml of IL-36α or IL-36γ for the indicated pre-determined times. Expression of phosphorylated (p-) and total IκBα was sequentially evaluated by Western blotting. The data are representative of three independent experiments. **(B)** SEMFs were transfected with NF-κBp65 siRNA and cultured for 2 days. The cells were then stimulated with or without 100 ng/ml of IL-36α (top panels) or IL-36γ (bottom panels) for 24 h. The mRNA expression of IL-6 and the indicated chemokines was then evaluated using real-time PCR. The mRNA expression for IL-6 and chemokine was converted to a value relative to β-actin mRNA expression and presented as fold-increase relative to the results for medium alone (no stimulation). Data are expressed as means ± SD of four independent experiments. *P < 0.05, **P < 0.01; significant differences from the values for IL-36α or IL-36γ stimulation.

The transcription factor NF-κB plays critical roles in the mRNA expression of inflammatory mediators. To investigate whether IL-36α or IL-36γ induce the activation of NF-κB, we evaluated the effects of both IL-36α and IL-36γ on IκBα phosphorylation and total IκBα degradation in human colonic SEMFs using western blot analysis. As shown in Figure 6A, both IL-36α and IL-36γ induced the phosphorylation and degradation of IκB. We also examined the involvement of NF-κB activation in the induction of IL-6 and CXC chemokines (CXCL1, CXCL2, and CXCL8) by IL-36α or IL-36γ using a siRNA specific for NF-κBp65. Blockade of the expression of NF-κBp65 significantly suppressed the induction of IL-6 and CXC chemokines (CXCL1, CXCL2, and CXCL8) by IL-36α or IL-36γ (Figure 6B) [we confirmed that the gene expression of NF-κBp65 was suppressed by the transfection of siRNA specific for NF-κBp65 using real-time PCR (Figure S2 in Supplementary Material)]. These results indicated that the activation of NF-κB is involved in the induction of proinflammatory mediators by both IL-36α and IL-36γ.

## Discussion

In the present study, we investigated the biological activity and the intracellular signal transduction of IL-36α and IL-36γ in human colonic SEMFs. We demonstrated that both IL-36α and IL-36γ are strong inducers of proinflammatory mediators, such as IL-6 and CXC chemokines (CXCL1, CXCL2, and CXCL8), and that MyD88 adaptor proteins were essential for the signal transduction of both IL-36α and IL-36γ. Furthermore, both IL-36α and IL-36γ induce proinflammatory mediators through the activation of MAPKs and NF-κB.

It has been reported that IL-36 cytokines play an important role in some human disorders, such as of psoriasis (16–18), rheumatoid arthritis (21–23), or pulmonary disease (24, 25). However, there is little information regarding the function of IL-36 in inflammatory response in the intestine. We, therefore, investigated the biological activities of IL-36 using human colonic SEMFs. We found that both IL-36α and IL-36γ strongly induce the production of IL-6 and CXC chemokines (CXC1, CXCL2, and CXCL8) in human colonic SEMFs. IL-6 has been reported to be an important factor in the acute phase response in various tissues via its broad proinflammatory actions (31), and chemokines direct the recruitment and migration of circulating leukocytes to inflammatory sites and determine the composition of leukocytes in inflammation (32–34). Thus, our results indicate that both IL-36α and IL-36γ contribute to the gut inflammation through the induction of cytokine and chemokine production from colonic SEMFs. Role of other member of IL-36 cytokine family, such as IL-36β, IL-38, and IL-36Ra (13), in the gut inflammation should be investigated in the future.

Various kinds of inflammatory mediators are involved in intestinal inflammation, such as IBD (33). For example, we have previously found that IL-17 expression is enhanced in the inflamed mucosa of IBD patients (35). Pathological role of TNF-α has been made clear by potent therapeutic effects of anti-TNF-α drugs (36, 37). So, we investigated the interaction between IL-36α/γ and IL-17A or between IL-36α/γ plus TNFα. We found that simultaneous stimulation of SEMFs with combination of IL-36α plus IL-17A or that of IL-36α plus TNFα synergistically upregulated the induction of inflammatory mediators. Similar results were detected by combination of IL-36γ plus IL-17A or that of IL-36γ plus TNFα. These data suggest that both IL-36α and IL-36γ may further enhance intestinal inflammation through synergism with IL-17A or TNFα via induction of proinflammatory mediators.

Receptors for IL-36 cytokines consist of IL-36R and the IL-1R accessory protein (IL-1RAcP). Following binding to the IL-36R, the IL-36/IL-36R complex recruits IL-1RAcP, leading to the activation of MAPK/NF-κB pathways (38). IL-36-mediated dimerization of IL-1RAcp with IL-36R drives productive signaling through engagement of cytoplasmic Toll/IL-1 receptor domains (13). However, the intracellular signaling pathways of IL-36 cytokines have not fully been identified in any cell type. In this study, we found that the activation of both MAPKs and NFκB is essential for the signal transduction of IL-36α or IL-36γ in SEMFs. Furthermore, we found that MyD88 adaptor proteins are critical for IL-36α- and/or IL-36γ-mediated induction of proinflammatory mediators.

In conclusion, we showed that both IL-36α and IL-36γ have a potential role as a strong inducer of the production of proinflammatory mediators by human colonic SEMFs. These responses were mediated by MyD88 signaling pathway followed by the activation of MAPKs and NFκB. IL-36 may play an important role in the inflammatory response in the colon and may be a new therapeutic target of IBD.

## Conflict of Interest Statement

The authors declare that the research was conducted in the absence of any commercial or financial relationships that could be construed as a potential conflict of interest.

## Supplementary Material

The Supplementary Material for this article can be found online at http://journal.frontiersin.org/article/10.3389/fmed.2015.00069
