# Supplementary material for: Interleukin(IL)-36α and IL-36γ Induce Proinflammatory Mediators from Human Colonic Subepithelial Myofibroblasts
Source: Front Med (Lausanne). 2015 Sep 22;2:69. doi: 10.3389/fmed.2015.00069 (PMC4585048; doi:10.3389/fmed.2015.00069)
Supplement: Supplementary file 1 [file Presentation_1.PDF]

Supplementary Material

Interleukin-36 Induces Proinflammatory Mediators from Human Colonic Subepithelial Myofibroblasts

Toshihiro Kanda[1], Atsushi Nishida[1*], Kenichiro Takahashi[1], Kentaro Hidaka[1], Hirotsugu Imaeda[1], Osamu Inatomi[1], Shigeki Bamba[1], Mitsushige Sugimoto[1], and Akira Andoh[1]

[1] Department of Medicine, Shiga University of Medical Science, Seta-Tsukinowa, Otsu, Japan

*Correspondence to:
Atsushi Nishida, MD, PhD
Department of Medicine, Shiga University of Medical Science, Seta-Tsukinowa, Otsu 520-2192, Japan
e-mail:atsuda@belle.shiga-med.ac.jp

Supplementary figure 1.

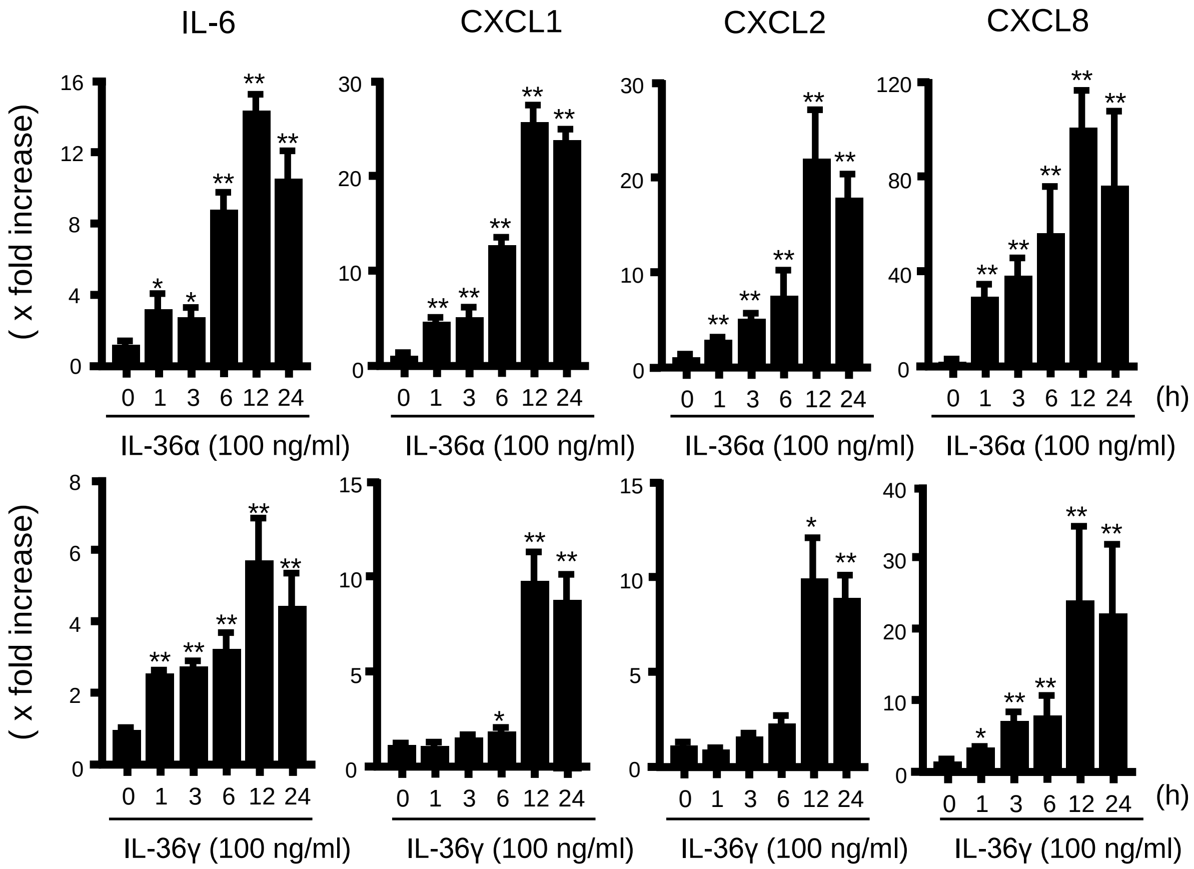

Supple Fig. 1. Kinetics of the mRNA expression of IL-6 and CXC chemokines induced by IL-36α and IL-36γ in human colonic SEMFs.

Human colonic SEMFs were stimulated with IL-36α (100ng/ml) or IL-36γ (100ng/ml) for predetermined times, and the mRNA expressions for IL-6 and CXC chemokines were determined using real-time PCR. Data were expressed as means ± SD of four independent experiments. **P* <0.05, ***P* <0.01; significant differences compared with the results at start.

Supplementary Figure 2.

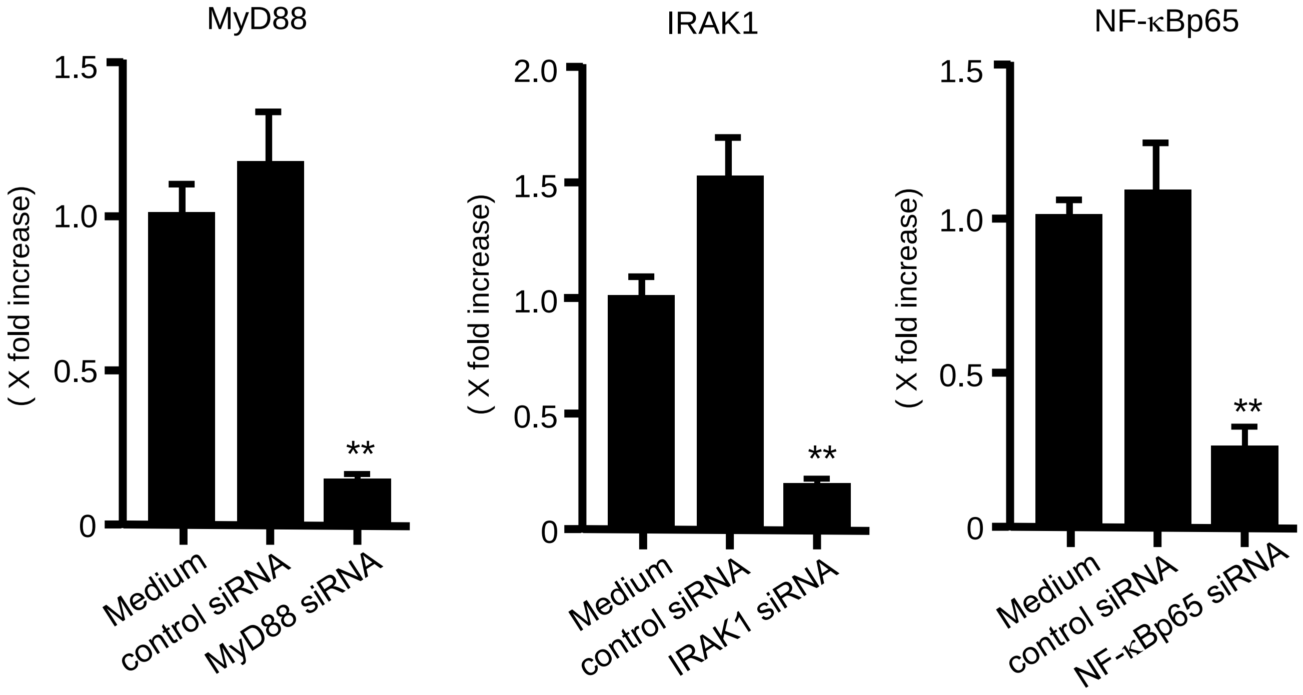

Supple. Fig. 2. Effect of siRNAs specific for MyD88, IRAK1 and NF-κB65 in human colonic SEMFs.

Human colonic SEMFs were transfected with siRNA specific for MyD88, IRAK1, and NF-κBp65. Control siRNA was also used. The effect of siRNAs was evaluated using real-time PCR. The gene expression of MyD88, IRAK1 and NF-κBp65 was significantly suppressed by specific siRNAs as compared to control siRNA. Data are expressed as means ± SD of three independent experiments. ***P* <0.01; significant differences compared with the results of control siRNA.
